# Anti-High Mobility Group Box 1 Neutralizing-Antibody Ameliorates Dextran Sodium Sulfate Colitis in Mice

**DOI:** 10.3389/fimmu.2020.585094

**Published:** 2020-10-30

**Authors:** Liping Chen, Junhua Li, Zhenghao Ye, Binghua Sun, Lu Wang, Yu Chen, Jian Han, Meiping Yu, Ying Wang, Qi Zhou, Ursula Seidler, De’an Tian, Fang Xiao

**Affiliations:** ^1^ Department of Gastroenterology, Tongji Hospital, Tongji Medical College, Huazhong University of Science and Technology, Wuhan, China; ^2^ Department of Nephrology, Tongji Hospital, Tongji Medical College, Huazhong University of Science and Technology, Wuhan, China; ^3^ Institute of Organ Transplantation, Tongji Hospital, Tongji Medical College, Huazhong University of Science and Technology, Wuhan, China; ^4^ Key Laboratory of Organ Transplantation, Ministry of Education, Wuhan, China; ^5^ Key Laboratory of Organ Transplantation, National Health Commission, Wuhan, China; ^6^ Key Laboratory of Organ Transplantation, Chinese Academy of Medical Sciences, Wuhan, China; ^7^ Department of Pathology, Tongji Hospital, Tongji Medical College, Huazhong University of Science and Technology, Wuhan, China; ^8^ Department of Gastroenterology, Hannover Medical School, Hannover, Germany

**Keywords:** colitis, anti-high mobility group box 1 neutralizing-antibody, toll-like receptor 4, macrophage, colonic barrier

## Abstract

High mobility group box 1 (HMGB1) is a ubiquitous nuclear protein in mammals. When released into the extracellular space, it acts as a damage-associated molecular pattern. This study investigates whether increased HMGB1 levels are found in the intestinal mucosa of ulcerative colitis (UC) patients, and whether an anti-HMGB1 neutralizing-antibody (HnAb) can inhibit the intestinal inflammation elicited by dextran sulfate sodium (DSS) in mice. Because toll-like receptor 4 (TLR4) is implicated in HMGB1-mediated immune cell activation, DSS colitis was also elicited in TLR4-deficient mice in the presence and absence of HnAb. The expression of HMGB1 in UC patients was examined. HnAb was administered *via* intraperitoneal injection to TLR4 deficient mice and their wild-type littermates, both being induced to colitis with DSS. Finally, the protective effect of HnAb and TLR4 deficiency were evaluated. In UC patients, HMGB1 was up-regulated in the inflamed colon. When administered during DSS application, HnAb alleviated the severity of colitis with a lower disease activity index, limited histological damages, and reduced production of proinflammatory cytokines. This antibody also limited colonic barrier loss, decreased colonic lamina propria macrophages and partially reversed the DSS treatment-associated dysbiosis. The protective effect of this antibody was enhanced in TLR4-deficient mice in some aspects, indicating that both additional HMGB1-mediated as well as TLR4-mediated inflammatory signaling pathways were involved in the induction of colitis by DSS. HnAb ameliorated colitis *via* macrophages inhibition and colonic barrier protection. It may therefore be a novel treatment option in colitis.

## Introduction

Inflammatory bowel diseases (IBD) represents a group of chronic relapsing inflammatory disorder, including Crohn’s disease and ulcerative colitis (UC). An increasing incidence of IBD is seen worldwide (1). Besides conventional therapy, anti-cytokine agents have been applied for IBD therapy. While initially highly efficient in many patients, the efficacy of biologics in inducing and maintaining clinical remission of IBD over extended period of times remains below 50% of treated patients (2, 3). Therefore, the identification of new therapeutic targets for the development of additional treatment option is of high clinical priority.

High mobility group box 1 (HMGB1) is a highly conserved nuclear protein in mammalian tissues, and is responsible for maintaining the structure of nucleosomes and regulating gene transcription (4). Activated immune cells release HMGB1 into the extracellular space, where it acts as an “alarmin” and accelerates the immune response. HMGB1 has been identified as a pro-inflammatory cytokine or damage-associated molecular pattern implicated in several inflammatory disorders, such as septic shock, rheumatoid arthritis, systemic lupus erythematosus, and recently in IBD (5). Expression of HMGB1 was elevated in serum, intestinal tissues or feces of patients and mouse models of IBD (6, 7), and HMGB1 was transported from nucleus to the cytoplasm during colonic inflammation (8, 9). Reducing HMGB1 level by probiotic supplementation in mice could partially ameliorate 2,4,6-trinitrobenzenesulfonic acid-induced murine colitis (10). HMGB1 was found to aggravate colonic inflammation by activating the immune response in colitis (11).

Among the receptors of HMGB1 such as toll-like receptors (TLR) TLR2, TLR4, TLR9, and receptor for advanced glycan end-products, TLR4 initiated signal transduction to yield inflammatory cytokines after binding to HMGB1 (12). Moreover, up-regulation of TLR4 could promote the progression of IBD (13).

In recent years, it is a hot issue to investigate the changes of colonic inflammation by inhibiting the function of HMGB1 using anti-HMGB1 antibody, ethyl pyruvate, glycyrrhizin, and other inhibitors (14–17). However, effective therapeutic alternatives based on the blockage of HMGB1-TLR4-associated immunological pathways to ameliorate colonic inflammation have not yet been developed. The purpose of this study was to investigate the protective effect of anti-HMGB1 neutralizing-antibody (HnAb) in dextran sulfate sodium (DSS)-induced colitis. Because TLR4 is an important mediator of HMGB1-induced amplification of the immune response, we tested the effect of HnAb also in mice that were deficient in TLR4.

## Materials and Methods

### Materials

RNA iso Plus, SYBR Premix Ex TaqTM and PrimeScript RT Master Mix for real-time PCR (RT-PCR) were procured from TaKaRa Biotechnology Inc. (Toyobo, Japan). Anti-myeloperoxidase (MPO) was bought from Thermo Fisher Scientific, Inc. (Waltham, MA, USA). Anti-HMGB1, anti-claudin-5, anti-occludin, anti-TLR4, and anti-MyD88 were acquired from Abcam Biotechnology Inc. (Cambridge, UK). Anti-ZO-1, anti-mucin 2 (sc-15334), and anti-NF-κB p65 were purchased from Santa Cruz Biotechnology Inc. (Santa Cruz, CA, USA). Their respective horseradish peroxidase-coupled secondary antibodies were obtained from KPL (Gaithersburg, MD, USA).

### Patients

UC patients with colonic inflammation restricted in the rectum were selected for this study. Upon having obtained consents from the patients, 14 pairs of inflamed intestinal samples and their corresponding adjacent non-inflamed intestinal samples were collected from patients with UC undergoing endoscopic biopsies at Tongji Hospital, Huazhong University of Science & Technology, Wuhan, China. Histopathology was confirmed by examining hematoxylin and eosin (HE)-stained tissue sections by qualified pathologists. UC diagnosis was established on the basis of conventional clinical, radiological, endoscopic, and histological findings against European Crohn’s and Colitis Organisation guidelines (18).

### Animals and Experimental Procedures

Female C57BL/6J [wild type (WT)] mice weighing 18–20g (8–10 w) were purchased from the Center for Disease Control and Prevention of Hubei Province (Wuhan, China). TLR4-/- mice (C57BL/10ScNJGpt, 8 weeks old) were obtained from GemPharmatech Co., Ltd, Nanjing, China. The mice were kept in specific pathogen-free (SPF) conditions. Acute colitis was induced by treating the animals with 4% DSS (36–50 kDa, MP Biomedicals, Solon, USA), given in the drinking water, for 7 days. Simultaneously, half of them were treated with HnAb, a polyclonal chicken IgY against murine HMGB1 neutralizing-antibody, (200 μg/mouse, Shino-Test Corporation, Tokyo, Japan) and others were treated with anti-IgY, a control chicken IgY antibody (200 μg/mouse, Shino-Test Corporation, Tokyo, Japan), *via* intraperitoneal injection on the 0th, 3rd, and 6th days of DSS feeding. Six groups with five mice per group were named WT, DSS-HnAb, DSS-IgY, TLR4-/- control, TLR4-/- DSS-HnAb, and TLR4-/- DSS-IgY, respectively. The mice were sacrificed on the eighth day and the full colons were removed.

### The Disease Activity Index and the Histological Examination

Mice were weighed and examined for diarrhea and rectal bleeding on daily basis. The DAI was scored according to the criteria previously described by Sann H et al. (19). DAI= score (weight loss + stool consistency + bleeding)/3. Blood in the feces was tested using a Hemoccult Assay Kit (Nanjing Jiancheng Bioengineering Institute, China). Body weight loss was calculated relative to day 1.

Colonic samples with formalin-fixed and paraffin-embedded were sectioned and hematoxylin and eosin-stained in standard procedures. Each section was graded by three blinded researchers. The colitis was histologically graded on a scale reported by Dieleman (20) and Soufli (21) with some modifications. Features of each section were graded in terms of, respectively, inflammatory cell infiltration (0-3), inflammation extent (0-3), and crypt damage (0-4). The degree of involvement was graded on a 0-4 scale. The grade for the feature then multiplies by the percentage involvement, reaching a score of inflammatory cell infiltration (0-12), inflammation extent (0-12), and crypt damage (0-16). Taken together, the total histological score of each section was obtained, ranging from 0 to 40 points (**Supplementary Table 1**).

### Real-Time PCR

The messenger RNA (mRNA) expression of HMGB1, interleukin-1β (IL-1β), tumor necrosis factor-α (TNF-α), interferon γ (IFN-γ), interleukin-6 (IL-6), interleukin-8 (IL-8), ZO-1, claudin-5, occludin, inducible nitric oxide synthase (iNOS), arginase 1 (Arg1), major histocompatibility complex II (MHC-II), receptor for advanced glycation end products (RAGE), TLR2, TLR4, TLR9, chemokine (C-X-C motif) receptor 4 (CXCR4), and MyD88 were detected by RT-PCR. First, total RNA was extracted from the intestinal samples by using TRIzol reagent. Then complementary DNA (cDNA) was synthesized by employing reversed transcriptional kit. For RT-PCR, x µg of cDNA template was added into a 25-µl reaction system, including with 1.0 µl of each primer (200 nM) and 12.5 µl of SYBR Premix ExTaq II. All primers were synthesized by Tsingke Biological Technology Co., Ltd. (Wuhan, China) (**Supplementary Table 2**). RT-PCR data were analyzed by utilizing the 2^−ΔΔCT^ method with β-actin as the reference gene.

### Western Blotting

Protein was extracted from the colonic tissues. Protein concentrations in the lysates were quantitatively determined by using a BCA Protein Assay Kit according to the manufacturer’s instructions. Intensity of the bands was quantified using IPP 6.0 software package with histone H3 and glyceraldehyde-3-phosphate dehydrogenase (GAPDH) as reference. The following antibodies were used: HMGB1 (1:1,000), TLR4 (1:500), MyD88 (1:1,000), claudin-5 (1:1,000), occluding (1:2,000), ZO-1 (1:500), GAPDH (1:10,000), histone H3 (1:3,000).

### Flow Cytometry

The colonic tissues were cut into small pieces by scissors, and then processed in a digestion buffer and Miltenyi gentleMACS Dissociator following by instructions. Homogenized intestinal tissues were passed through a 40-μm nylon mesh to get a single-cell suspension. Cells were stained with F4/80, CD11b and CD86, CD11c and CD206 antibodies (eBioscience, San Diego, USA) and incubated for 20 min at 4°C in the dark. Macrophages were identified with CD11b+ F4/80+ and CD86+ macrophages were identified with CD11b+ F4/80+ CD86+. After washing, macrophage cell subsets were collected and flow cytometrically analyzed (FACScalibur Flow Cytometer, Becton-Dickinson Immunocytometry Systems, San Jose, USA). Cell viability was preformed using Aqua Dead Cell Stain Kit (L34965, Thermo Fisher Scientific, USA). The percentage of cells in live/singlets gate was evaluated by the number of live cells to get an absolute live-cell count. At least 10, 000 live events were accumulated through forward- and side-scatter parameters. Cell sorting was processed on a FACSAria II instrument (BD Biosciences, San Jose, USA) with the same configuration as the LSR II. Cytospins were prepared from the sorted cells and cell populations were identified using a sequential gating strategy. Data were analyzed by using FlowJo software (version 7.01, Tree Star Software, San Carlos, USA).

### Transmission Electron Microscopy

Transmission electron microscopy was used to detect alterations in tight junction ultrastructure and macrophage infiltration. Specimens from the intestinal tissues were washed three times in 0.1 mol/l cacodylate buffer and then immersed in 2.5% glutaraldehyde for 12 h, post-fixed in 1% osmium tetroxide for 1 h. Then the ultrathin sections were prepared and mounted on copper girds, double staining with uranyl acetate and citrate acid, and observed under a transmission electron microscope (JEM-1200 EX II TEM, JEOL, Tokyo, Japan) operated at 80 kV after dehydration and embedding.

### Immunohistochemistry

The expression of MPO, HMGB1 was immunohistochemically detected by using a Super Sensitive TM IHC Detection System Kit (Bioworld Technology Inc. Louis Park, MN, USA). Tissue slices were immersed in heated sodium buffer (pH 9.0) for antigen retrieval. Then tissue slices were sequentially incubated with 3% hydrogen peroxide, 5% normal goat serum, and the primary antibody against HMGB1 (1:2,000, Abcam, Cambridge, UK) or MPO (1:1,000, Thermo Fisher Scientific, Rockford, USA). The antibody signal was evaluated by incubated with secondary antibody conjugated to horseradish peroxidase. The slices were examined under a light microscope equipped with a built-in DC750 digital camera system (Leica Microsystems, Wetzlar, Germany). Each slice was semiquantitative graded by two pathologists according to a scale described by McCarty KS et al. (22). The algorithm for the histochemical score (histoscore) was: histoscore =∑(*i*+1) × *Pi*, where *i* = intensity of staining (0-4) and *Pi* = percentage of cells stained at each intensity.

### Immunofluorescence

The changes in mucus barrier integrity were assessed by immunofluorescence analysis. Frozen intestinal tissues were minced into 10-µm-thick fragments and stuck to slides. Slides were incubated with rabbit anti-mouse tight junction proteins or E-cadherin antibodies. Then incubated with Alexa 488-conjugated secondary antibody for 1 h at room temperature. Afterwards, the nuclei were stained with DAPI solution for 1 min at room temperature. Immunoﬂuorescence images were taken under a confocal microscope (Olympus FV-1000, Olympus, Tokyo, Japan). All experiments were repeated at least three times with different batches of samples.

### 16S Ribosomal RNA Sequencing and Analysis

Colonic mucosal biopsy scraping was taken from the distal colon using cold biopsy forceps. Non-adherent fecal material was removed by washing and swirling in 1 ml sterile PBS. The mucosal samples were then flash-frozen at −80°C for further microbiota analysis. Genomic DNA from mucosal samples was extracted using the protease K lysis and phenol chloroform extraction. 16S rRNA genes were amplified by PCR using general bacterial primers (515F 5’-GTGCCAGCMGCCGCGGTAA-3’ and 926R 5’-CCGTCAATTCMTTTGAGTTT-3’). The raw fastq files were demultiplexed according to the barcode, as described by Li XX et al. (23). Using the UPARSE pipeline (http://drive5.com/usearch/manual/uparsecmds.html), the demultiplexed reads were clustered into operational taxonomic units (OTUs) at 97% sequence identity. OTU taxonomies (from Phylum to Species) were determined on the basis of NCBI. For the alpha-diversity analysis, Shannon, Simpson, Chao, ACE index were calculated using mothur (version 1.33.3, http://www.mothur.org/) and the corresponding curves were plotted by R (version 3.2.3, R Development Core Team, 2016). For beta-diversity metrics, the weighted and unweighted UniFrac distance matrix were calculated by mothur, and visualized with tree and principal coordinate analysis (PCoA) by R.

### Statistics

*Data were expressed as mean ± SEM.* Data between groups of human samples were analyzed by paired Student’s *t* test. Data among groups of mice were assessed by one-way ANOVA or two-way ANONA, followed by Tukey’s post-test using GraphPad Prism (version 7.01, GraphPad Prism Inc., USA). A two-side *P* < 0.05 was considered statistically significant.

### Ethics Statement

The patient study was performed following the guidelines of the Ethics Committee of the Tongji Hospital and conform to the ethical standards of the World Medical Association Declaration of Helsinki. Participants’ written informed consent was received prior to inclusion in the study. All animal experiments were approved by the Animal Care and Use Committee of Tongji Hospital, Huazhong University of Science and Technology.

## Results

### Elevated HMGB1 Expression in Inflamed Colonic Tissues of Ulcerative Colitis Patients

**Figure 1:**
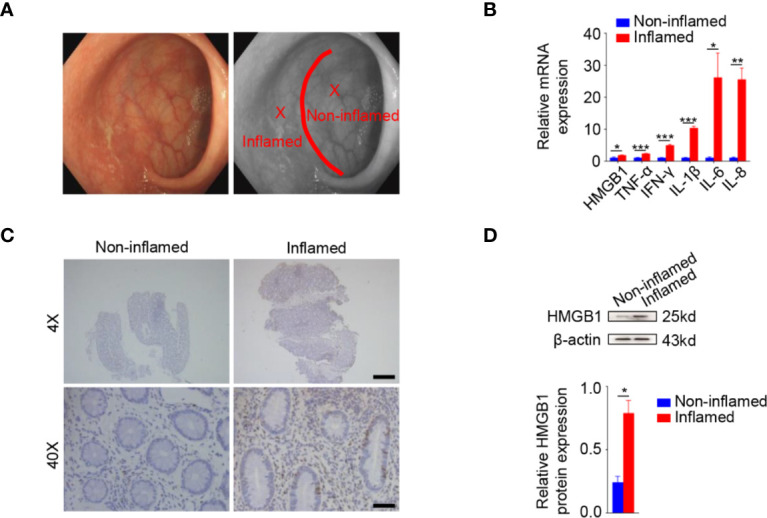
Increased HMGB1 in the inflamed colonic tissues of ulcerative colitis (UC) patients. **(A)** Rectal inflamed biopsies and their adjacent non-inflamed samples of ulcerative rectitis patients. Ulcerative rectitis endoscopy (left panel). Schematic diagram of inflamed and non-inflamed rectal biopsies in ulcerative rectitis patients during endoscopy (right panel). Red line, demarcation line of inflamed and non-inflamed area. X, biopsy point. **(B)** Relative messenger RNA (mRNA) expression of HMGB1, TNF-α, IFN-γ, IL-1β, IL-6, and IL-8 mRNA were significantly increased in the inflamed tissues compared to the non-inflamed tissues. Glyceraldehyde-3-phosphate dehydrogenase (GAPDH) was used as an internal control. **(C)** Immunohistochemistry (IHC) staining of HMGB1 in non-inflamed (left panels) and inflamed (right panels) tissues. Scale bar: 400 μm (upper panels), 40 μm (lower panels). **(D)** Western analysis demonstrated significantly higher HMGB1 protein expression in the inflamed tissues compared to the non-inflamed tissues. Data in **(A)** were representative of 14 independent experiments. Data in **(B, D)** were presented as mean ± SEM of 14 independent experiments. *P < 0.05, **P < 0.01, ***P < 0.001, by paired Student’s *t* test **(B, D)**.

In order to determine HMGB1 expression during colonic inflammation, 14 patients with UC [aged 46.10 ± 1.01 years, 9 (64.3%) males] were recruited. The patients were all E1 according to Montreal classification and their Mayo score was 4.93 ± 0.07. Fourteen pairs of tissue samples were endoscopically biopsied from the inflamed rectal areas and their adjacent non-inflamed regions. Inflamed and non-inflamed regions in the rectum of UC patients were shown in a representative endoscopic picture (**Figure 1A**). A schematic diagram was drawn to indicate the inflamed and non-inflamed regions as well as the biopsy sites (**Figure 1A**). The mRNA expression of TNF-α, IFN-γ, IL-1β, IL-6, and IL-8 was significantly increased in the inflamed tissues compared to adjacent non-inflamed tissues, which was consistent with the endoscopic manifestations. In line with the changes in these inflammatory cytokines, the mRNA expression of HMGB1 was also increased in inflamed regions (**Figure 1B**).

To determine the protein expression of HMGB1 in the inflamed and adjacent non-inflamed samples, we performed immunohistochemical analysis and western blotting. Immunohistochemical results showed HMGB1 increased in inflamed tissues compared to their non-inflamed counterparts (**Figure 1C**). Western blotting further confirmed the elevated HMGB1 protein in inflamed tissues as compared to the adjacent non-inflamed tissues in UC patients (**Figure 1D**).

### HnAb Treatment Attenuates the Severity of Dextran Sulfate Sodium-Induced Colitis

**Figure 2:**
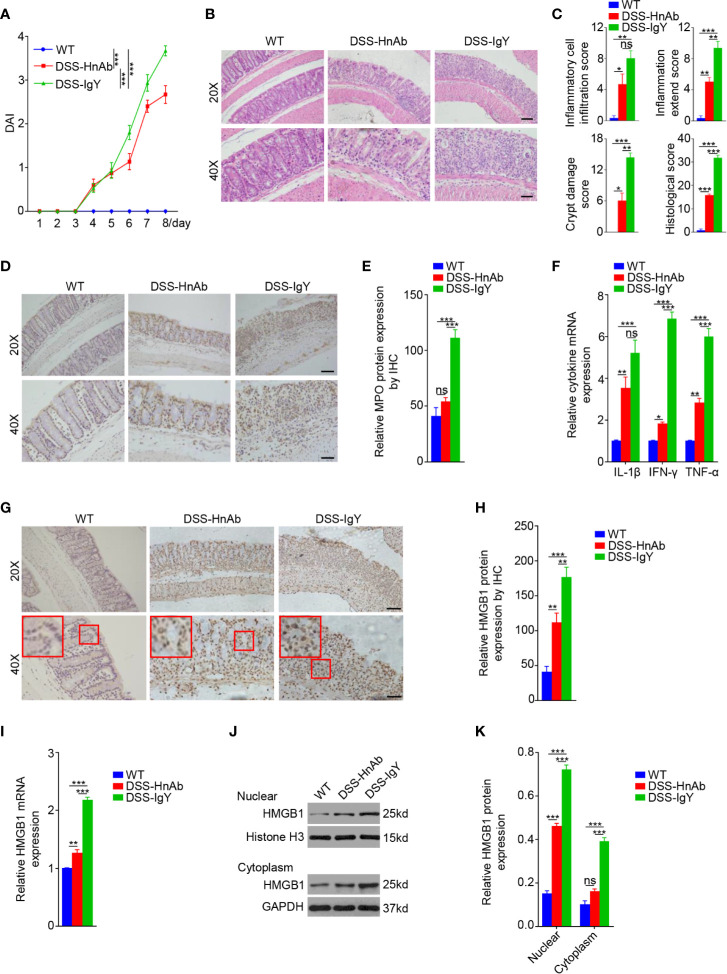
HnAb treatment attenuates the severity of dextran sulfate sodium (DSS)-induced colitis mice. **(A)** The disease activity index (DAI) gradually increased from day 4 onward in the HnAb-treated and vehicle treated DSS-treated mice. **(B)** Histological examination displays colitis severity. Scale bar: 80 μm (upper panels), 40 μm (lower panels). **(C)** Histological grading of colitis on day 8. There was a significant difference of scores when DSS/IgY-treated mice *vs.* the control, DSS/IgY-treated mice *vs.* DSS/HnAb-treated mice, DSS/HnAb-treated mice *vs.* the control. **(D)** The expression of MPO was displayed by immunohistochemistry (IHC). Scale bar: 80 μm (upper panels), 40 μm (lower panels). **(E)** The expression levels of MPO in the colon were evaluated by IHC using a semi-quantitative scoring system (see *Materials and Methods* section). A significant difference between the scores of DSS/IgY-treated mice *vs.* the control, DSS/IgY-treated mice *vs.* DSS/HnAb-treated mice. No significant difference was found between DSS/HnAb-treated and control mice. **(F)** The messenger RNA (mRNA) expression levels of IL-1β, IFN-γ, and TNF-α in the colonic tissues of the three groups were detected by RT-PCR. The expression of these three cytokines was increased in the colonic tissues of colitis mice, and HnAb administration resulted in reduced expression level. **(G)** The expression of HMGB1 was assessed by IHC. Scale bar: 80 μm (upper panels), 40 μm (lower panels). **(H)** Semiquantiative score for HMGB1 expression by IHC. A significant difference was observed between HMGB1 expression in DSS/IgY-treated *vs.* control colonic tissue, DSS/IgY-treated *vs.* HnAb treated colonic tissue, and DSS/HnAb-treated *vs.* control colonic tissue. **(I)** HMBG1 mRNA expression levels were detected by RT-PCR. The expression of HMGB1 was increased in the colonic tissues of colitis mice, and HnAb administration resulted in reduced expression level. **(J, K)** HMGB1 levels in nuclear and cytoplasmic lysate detected by Western blotting and semiquantiative scoring as described in the *Materials and Methods*. HMGB1 level was reduced in nucleus and cytoplasm of DSS/HnAb-treated compared to DSS/IgY treated colon. Data in **(B, D, G, J)** were representative of 5 independent experiments. Data in **(A, C, E, F, H, I, K)** were presented as mean ± SEM of 5 independent experiments. *P < 0.05, **P < 0.01, ***P < 0.001, ns, not significant, by two-way ANOVA with Tukey’s post-test **(A)**, by one-way ANOVA with Tukey’s post-test **(C, E, F, H, I, K)**.

HnAb was administrated into mice with DSS-induced colitis to investigate the effect of HMGB1 inhibition on colitis severity. HnAb-treated mice delayed the manifestation of fecal occult blood and diarrhea, relieved weight loss, thus contributing to a significantly decreased DAI of mice at day 8 compared with the IgY-treated DSS-induced colitis mice (**Figure 2A**, **Supplementary Figure 1**). The histological score was significantly decreased after HnAb administration compared with vehicle-treated DSS-induced colitis mice (**Figures 2B, C**).

Myeloperoxidase (MPO) expression was examined to evaluate the neutrophils accumulation in colonic tissues. Semiquantitative immunohistochemistry (IHC) suggested that MPO expression was increased significantly in colonic tissues of DSS treated mice comparing to the normal control. The administration of HnAb inhibited significantly the MPO expression as compared to vehicle-treated DSS-induced colitis mice (**Figures 2D, E**).

To further evaluate the severity of the inflammatory response, pro-inflammatory factors such as IL-1β, IFN-γ, and TNF-α in the colonic tissues were measured. The results showed that the mRNA expression of IL-1β, IFN-γ, and TNF-α in colonic tissues increased after DSS administration, and such increase was suppressed with respect to IFN-γ and TNF-α, but not to IL-1β, by HnAb treatment (**Figure 2F**).

The mRNA expression of HMGB1 dropped dramatically after the administration of HnAb compared to DSS-treated mice (**Figure 2I**). In order to evaluate whether HMGB1 was translocated from nucleus to cytoplasm and extracellular region in colitis, IHC, and Western blotting were performed. IHC exhibited that HMGB1 was released to cytoplasm and extracellular site in DSS-induced murine colon (**Figure 2G**). Administration of HnAb could partially block HMGB1 redistribution into the cytoplasm and extracellular region (**Figure 2G**). Semiquantitative IHC suggested that HMGB1 expression was inhibited significantly in colonic tissues of HnAb-treated mice as compared to vehicle-treated mice with DSS-induced colitis (**Figure 2H**).

Western blotting showed that HMGB1 expression in both the nucleus and cytoplasm were down-regulated after the administration of HnAb as compared to IgY-treated colitis mice. Nonetheless, nuclear and cytoplasmic HMGB1 was elevated in colonic tissues in DSS-colitis mice compared to the normal control mice (**Figures 2J, K**).

The findings indicated that, in the course of colitis, HMGB1 was transported from nucleus to cytoplasm and even extracellular region, and HnAb could hamper the redistribution of nuclear HMGB1 into cytoplasm.

Taken together, these data suggested that HnAb could prevent colitis from further deterioration.

### HnAb Treatment Ameliorates the Colonic Barrier Defect in Dextran Sulfate Sodium-Induced Colitis

**Figure 3:**
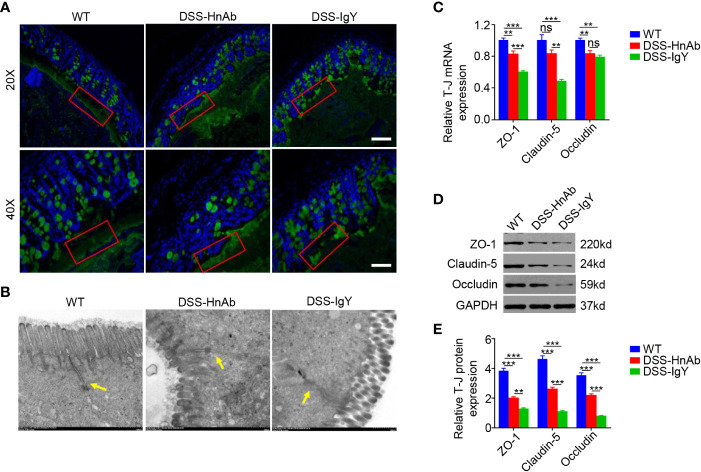
HnAb treatment enhances adherent mucus layer and tight junction integrity during dextran sulfate sodium (DSS) colitis induction. **(A)** The mucus layer was delineated by immunofluorescence staining for mucin 2. The mucus layer in DSS/HnAb-treated colon was relatively intact, while no complete mucus layer structure could be found in DSS/IgY-treated colon. Scale bar: 80 μm (upper panels), 40 μm (lower panels). **(B)** Tight junction structure was observed by transmission electron microscope. Tight junction structure was relatively intact in DSS/HnAb-treated comparing to DSS/IgY-treated colon. **(C)** A significant increase for both ZO-1 and claudin-5 mRNA levels, but not for occludin mRNA levels was seen in DSS/HnAb-treated compared to DSS/IgY-treated colon (*P*<0.05). **(D, E)** Likewise, a significant increase for ZO-1, claudin-5, and occludin was seen in DSS/HnAb-treated *vs.* DSS/IgY-treated colon by Western analysis. Data in **(A, B, D)** were representative of five independent experiments. Data in **(C, E)** were presented as mean ± SEM of five independent experiments. **P < 0.01, ***P < 0.001, ns, not significant, by one-way ANOVA with Tukey’s post-test **(C, E)**.

Mucin 2, one of the key mucins, was stained to delineate the mucus layer (**Figure 3A**). In this study, immunofluorescence staining revealed that an integral mucus layer consisted of a continuous and evenly distributed mucus layer between the interstinal epithelium and the intestinal lumen in normal control, while no structurally complete mucus layer could be found in DSS-treated animals. Upon administration of HnAb, the mucus layer was relatively intact and only a few fractures could be observed (**Figure 3A**).

The colonic epithelial barrier also plays an important role in colitis. A monolayer of epithelial cells and the tight junctions assembled by connexins are the pivotal components of the intestinal epithelial barrier. Tight junction structure was observed by transmission electron microscopy. Though no complete tight junction structure could be found after DSS administration, relatively intact tight junction structure could be observed in HnAb-treated colitis mice (**Figure 3B**). Furthermore, tight junction proteins zonula occludens-1 (ZO-1), claudin-5 and occludin were detected. The mRNA expression levels of ZO-1 (*P*<0.001) and claudin-5 (*P*<0.01), but not occludin (*P*=0.587), were significantly higher in colonic tissues of HnAb-treated DSS-induced colitis mice than IgY-treated DSS-induced colitis mice (**Figure 3C**). The protein expression levels of ZO-1, claudin-5, and occludin were markedly decreased in DSS-induced colitis mice than in normal controls but they were slightly increased after HnAb administration compared with vehicle (IgY)-treated DSS-induced colitis mice (**Figures 3D, E**).

These data indicated that HnAb could partially protect the colonic barrier from DDS-induced colonic damage.

### HnAb Treatment Decreased Macrophages in the Colonic Lamina Propria

**Figure 4:**
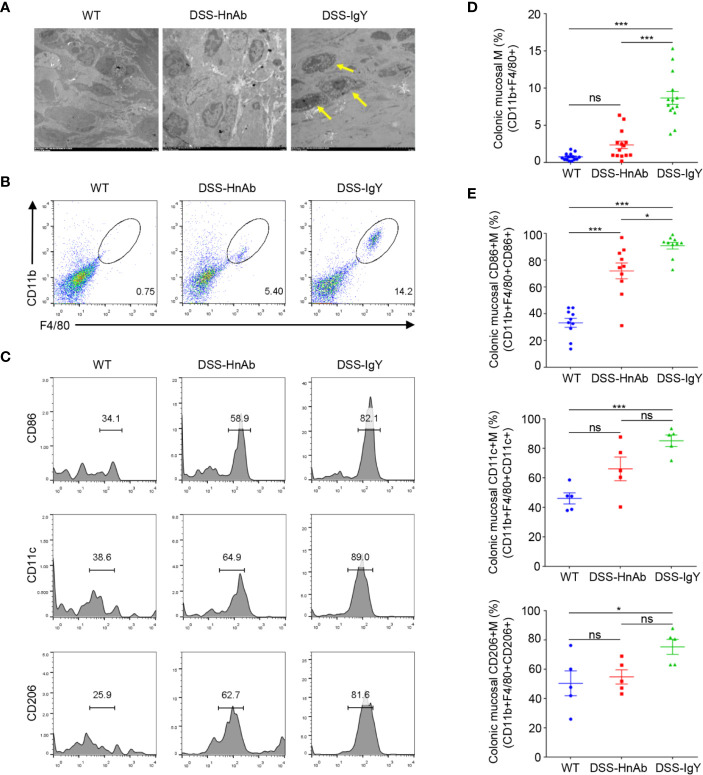
Macrophages and CD86+ macrophages are decreased in DSS/HnAb-treated colon. **(A)** Macrophages were observed in transmission electron microscopic images. Few macrophages could be found in lamina propria in the control, while an increased number was present in dextran sulfate sodium (DSS)/IgY con. DSS/HnAb treatment reduced the number of lamina propria macrophages. **(B)** Macrophages were identified by the high expression of F4/80 and CD11b. **(C)** Activation markers CD86, CD11c, CD206 were used to count macrophages by FACS analysis. **(D)** Number of macrophages in % of total counted cells was significantly higher in DSS/IgY treated *vs.* control colon (*P*<0.001) as well as *vs.* DSS/HnAb-treated colon (*P*<0.001), but not in DSS/HbAg-treated *vs.* control colon. **(E)** Significant increase of CD86+ macrophages, but not CD11c+ and CD206 macrophages in DSS/HbAg-treated *vs.* control colon. Data in **(A–C)** were representative of five independent experiments. Data in **(D, E)** were presented as mean ± SEM of five independent experiments. *P < 0.05, ***P < 0.001, ns, not significant, by one-way ANOVA with Tukey’s post-test **(D, E)**.

Transmission electron microscopy revealed much more macrophages in the colonic lamina propria of DSS-induced colitis mice than in the normal control. While fewer macrophages could be found after HnAb administration compared to IgY-treated DSS-induced colitis mice (**Figure 4A**).

To further correlate the reduced macrophages with the ameliorated colitis mice, we isolated lamina propria cells from colon. Macrophages were identified by the high expression of F4/80 and CD11b (**Figure 4B**). Then activation markers CD86, CD11c, CD206 were used to identified and gated for macrophages, respectively (**Figure 4C**). The percentage of macrophages in total counted cells was significantly higher in DSS/IgY treated *vs.* control colon (*P*<0.001) as well as *vs.* DSS/HnAb-treated colon (*P*<0.001), but not in DSS/HnAg-treated *vs.* control colon (**Figure 4D**). Significant increase in percentage of CD86+, but not CD11c+ and CD206 macrophages in DSS/HbAg-treated *vs.* control colon (**Figure 4E**).

### HnAb Treatment Suppresses TLR4-Myeloid Differentiation Factor 88 Expression in Dextran Sulfate Sodium-Induced Colitis

**Figure 5:**
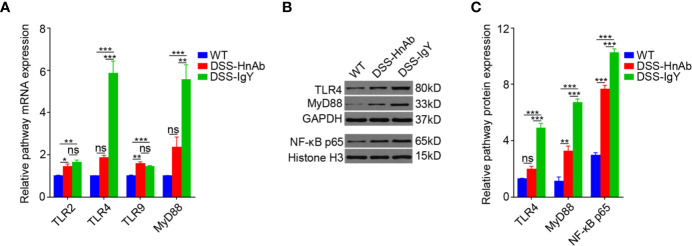
Concomitant dextran sulfate sodium (DSS)/HnAb treatment attenuates TLR4 and MyD88 upregulation during DSS induction of colitis. **(A)** Significant decrease for both MyD88 and TLR4 messenger RNA (mRNA), but not for TLR2 and TLR9 when HnAb treatment comparing to IgY treatment for DSS-induced colitis mice. **(B, C)** TLR4 and MyD88 protein was also dramatically reduced in DSS/HnAb-treated *vs.* DSS/IgY-treated colonic tissue (*P* < 0.001). Data in **(A, C)** were presented as mean ± SEM of five independent experiments. *P < 0.05, **P < 0.01, ***P < 0.001, ns, not significant, by one-way ANOVA with Tukey’s post-test **(A, C)**.

The receptors of HMGB1 have been found to be mainly trans-membrane receptors such as TLR2, TLR4, and TLR9. MyD88, as an important adaptor protein, plays a key role in HMGB1 pathway. This study showed that expression of TLR2, TLR4, TLR9, and MyD88 mRNA was significantly increased in colonic tissues of DSS-induced colitis mice relative to the normal control mice. However, HnAb treatment decreased the mRNA expression of TLR4 and MyD88, but not that of TLR2, TLR9, compared to vehicle-treatment in DSS-induced colitis mice (**Figure 5A**). TLR4 receptor might be one of the major receptors for HMGB1 in DSS-induced colitis.

Protein expression of both TLR4 and MyD88 was significantly elevated in colonic tissues of DSS-induced colitis mice compared to the normal control mice by Western blotting. The expression of both TLR4 and MyD88 was significantly down-regulated in HnAb treated colitis mice relative to vehicle-treated DSS-induced colitis mice (**Figures 5B, C**).

Furthermore, detection of the representative protein of NF-κB p65 revealed that the expression of NF-κB p65 was significantly up-regulated in colonic tissues of DSS-induced colitis mice as compared to the normal control mice, while HnAb treatment significantly decreased the expression of NF-κB p65 as compared to the vehicle treatment in DSS-induced colitis mice (**Figures 5B, C**).

### HnAb Treatment Further Ameliorates Colonic Inflammation in TLR4-/- Mice

**Figure 6:**
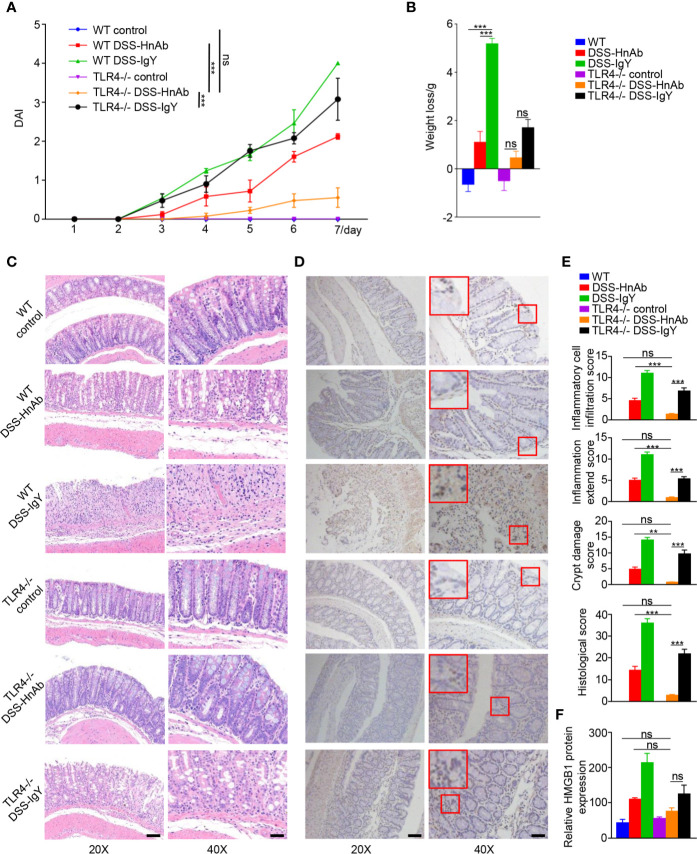
TLR4 deficiency and HnAb treatment haven an additive effect in the attenuation of dextran sulfate sodium (DSS)-induced colitis. **(A)** Time course of DAI increase during DSS colitis induction in wild type (WT) and TLR4-/- mice with and w/o concomitant HnAB treatment. **(B)** Body weight loss was decreased with TLR4 deficiency. No significant decrease of body weight loss was found after HnAb-treated DSS-induced colitis comparing with the control in TLR4-/- mice. **(C)** Mucosal damage shown by HE staining in the different genotype/treatment groups. Scale bar: 80 μm (left panels), 40 μm (right panels). **(D)** Immunohistochemistry (IHC) HMGB1 expression in the different genotype/treatment groups. Scale bar: 80 μm (left panels), 40 μm (right panels). **(E)** Lowest histological score in DSS/HnAb-treated TLR4-/- mice, followed by DSS/HnAb-treated WT colon. Lower histological score in DSS/IgY-treated TLR4-/- compared to DSS/IgY-treated WT colon. **(F)** IHC staining showed that HMGB1 was released to cytoplasm and extracellular site in DSS-induced mice colon. Administration of HnAb could partially block HMGB1 redistribution into extracellular region. Data in **(C, D)** were representative of five independent experiments. Data in **(A, B, E, F)** were presented as mean ± SEM of five independent experiments. **P < 0.01, ***P < 0.001, ns, not significant, by two-way ANOVA with Tukey’s post-test **(A)**, one-way ANOVA with Tukey’s post-test **(B, E, F)**.

A significantly decreased DAI was observed after HnAb application compared to IgY application in DSS-treated TLR4-/- mice at day 7. Regarding the protective effect on DAI, HnAb application in DSS-treated TLR4-/- mice was remarkably stronger than HnAb application in DSS-treated WT mice. No significant difference in DAI was found between DSS-induced TLR4-/- mice treated by HnAb and normal control WT mice (**Figure 6A**). HnAb-treated mice partially relieved weight loss (**Figure 6B**).

Histological score was decreased after HnAb administration as compared with vehicle-treated DSS-induced colitis mice. The histological score was further reduced in TLR4-/- mice as compared to their WT littermates. No significant difference in histological score was found between DSS-treated TLR4-/- mice by HnAb application and normal control WT mice (**Figures 6C, E**).

IHC showed that HMGB1 was released to cytoplasm and extracellular sites in DSS-induced colitis. Administration of HnAb could partially block HMGB1 redistribution into cytoplasm and extracellular region. The expression of HMGB1 was decreased in TLR4-/- mice. No significant difference in HMGB1 protein expression was found between DSS-treated TLR4-/- mice by HnAb application and normal control WT mice (**Figures 6D, F**).

There existed no significant differences in both ZO-1 and occludin protein expression levels between DSS-treated TLR4-/- mice by HnAb application and normal control WT mice, though claudin-5 protein expression was significantly lower in colonic tissues of DSS-treated TLR4-/- mice by HnAb application than normal control WT mice (**Supplementary Figure 2**). The expression of M1/M2 markers iNOS, Arg1, MHC-II, and the pro-inflammatory cytokines TNF-α, IL-6, IL-1β was explored by RT-PCR. Decreased mRNA expression levels for iNOS, Arg1, MHC-II, IL-1β, IL-6, TNF-α were found after HnAb application in DSS-treated WT and TLR4-/- mice (**Supplementary Figure 3**).

Further, molecular pathway of HMGB1 was explored by testing the expression of molecules such as RAGE, TLR2, TLR4, TLR9, CXCR4, which were the main molecules interaction with HMGB1. Decreased mRNA expression levels for not only TLR4 and MyD88, but also CXCR4, was found after HnAb application *vs.* IgY application in DSS-treated WT mice. Decreased CXCR4 mRNA expression was also detected when HnAb application *vs.* IgY application in DSS-treated TLR4-/- mice. (**Supplementary Figure 4**).

These data indicated that TLR4 deficiency could enhance the protective effect of HnAb on colonic inflammation.

### Microbiota Changes Are Partially Reserved by HnAb Treatment in Mice

**Figure 7:**
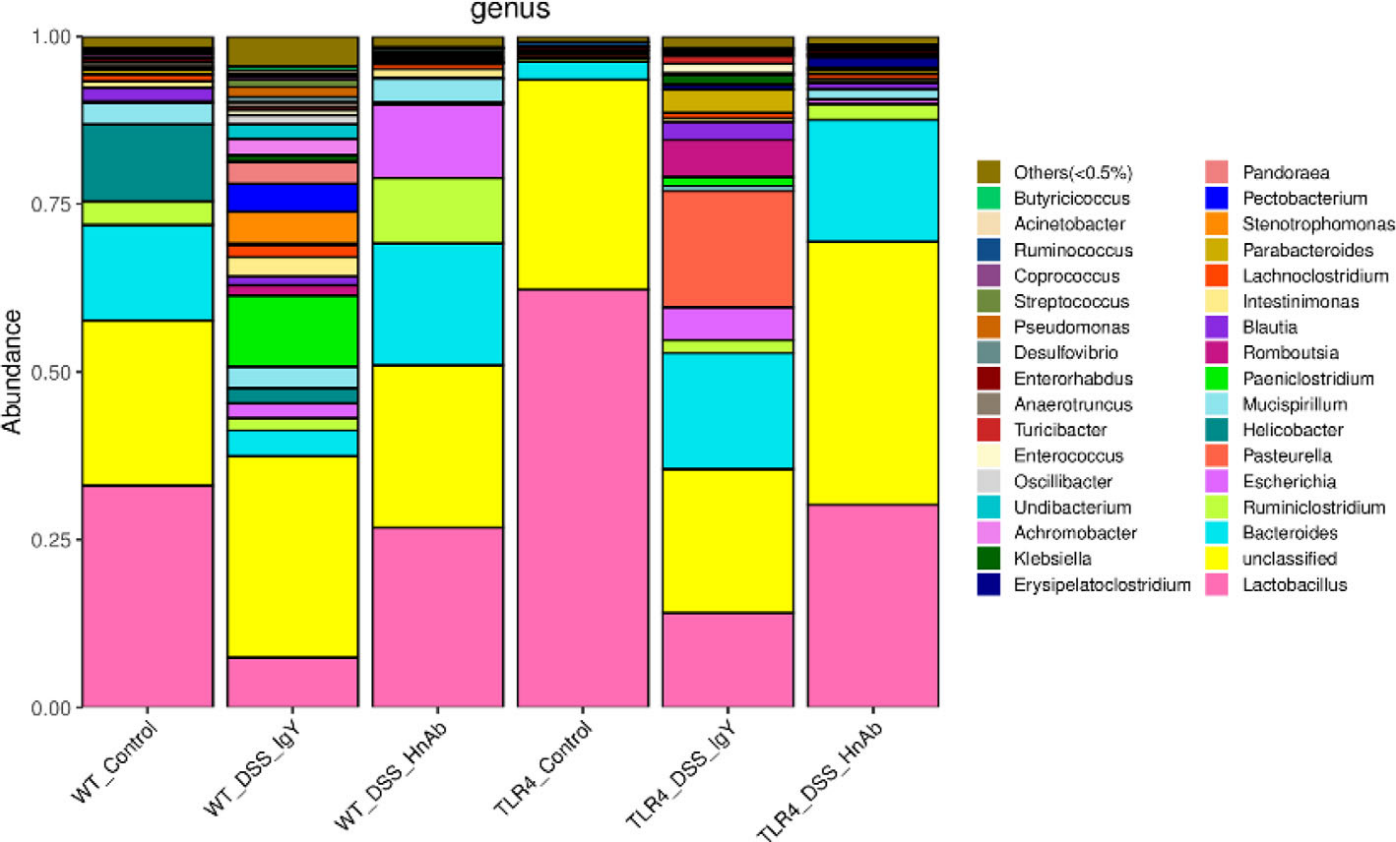
Microbiota changes are partially reserved at genus level by HnAb treatment in dextran sulfate sodium (DSS)-induced colitis mice. Compared with wild type (WT) normal control group, DSS treatment decreased proportion of *Lactobacillus*, whereas HnAb treatment could partially reverse microbiota pattern towards the normal group. In TLR4-/- mice, such reversion by HnAb treatment was more apparent.

To evaluate whether HnAb-induced alterations in colonic inflammation were related to the alteration of the gut microbial ecosystem, total mucosal microbiota profiles from mice were analyzed by 16S rRNA sequencing. Compared with WT normal control group, decreased proportion of *Lactobacillus* were observed in DSS-induced colitis mice, whereas HnAb treatment could partially reverse microbiota pattern towards the normal group. In TLR4-/- mice, such reversion by HnAb treatment was not more apparent (**Figure 7**). There existed no significant differences among the six groups in terms of Shannon and Simpson Index. To further investigate the effects induced by HnAb treatment, we employed linear discriminant analysis effect size (LEfSe) to evaluate the changed bacterial taxa (from phylum to species) among the six groups. The dominating taxa in WT normal control group at the genus level were enriched in *Lactobacillus*, which decreased after DSS administration. The changes were partially reversed by HnAb treatment. Meanwhile, TLR4 deficiency decreased the abundance of *Bacteroides*, *Helicobacter*, *Ruminiclostridium*, and increased the abundance of *Lactobacillus* compared to corresponding WT group (**Figure 7**, **Supplementary Figure 5**).

## Discussion

HMGB1, a nuclear non-histone protein, plays a critical role in the immune response (24). It can be actively secreted by activated immune cells like macrophages under certain specific conditions, or passively released from damaged or necrotic cells (25). When released from cells, HMGB1 induces an inflammatory response through a variety of receptor-mediated signal transduction pathways, such as the TLR4/MyD88/NF-κB pathway (26). After NF-κB activation, the release of pro-inflammatory factors including IL-1β, IFN-γ, and TNF-α is markedly increased, thus promoting the synthesis and release of HMGB1 in immune cells including macrophages (27).

In this study, the concomitant application of HnAb reduced the intestinal inflammatory damage induced by DSS, assessed by the colitis score, MPO expression and the expression levels of the cytokines IL-1β, IFN-γ, TNF-α in colonic tissues. It also ameliorated disease activity, indicated by lower DAI in HnAb-treated DSS-exposed mice compared to DSS-exposed sham-treated mice. HnAb antagonizes the activity of HMGB1 and hinders the effect of endogenous HMGB1. In recent years, the protective effect of HMGB1 inhibition on colitis has gradually attracted an attention. Inhibitors of HMGB1 currently mainly contain anti-HMGB1 antibody, ethyl pyruvate, and other inhibitors including glycyrrhizin, DNA-binding sphere based on the high nucleophilicity of HMGB1 and HMGB1 A box. The protective effect of HnAb was similar to that observed in previous studies (11, 28, 29). Roberta V and his colleagues found cytoplasmic HMGB1 expression was significantly enhanced in the inflamed tissues *vs.* uninflamed tissues of the IBD patients, which indicting HMGB1 was transported from nucleus to the cytoplasm during colonic inflammation (8). Our study also detected increased cytoplasmic HMGB1 expression.

HMGB1 release from damaged tissue accelerates macrophages infiltration (30) and macrophages promote further HMGB1 release (31), leading to a vicious cycle and increased inflammatory tissue damage (32). In this study, HnAb treatment decreased the colonic infiltration with lamina propria macrophages, especially CD86+ macrophages, in DSS-treated mice. Our study found M1/M2 markers iNOS, Arg1, MHC-II, and the pro-inflammatory cytokines TNF-α, IL-6, IL-1β were down-regulated after HnAb application in DSS-treated WT and TLR4-/- mice. It indicated TLR4 deficiency and HnAb treatment had an effect in activation of macrophages. Since macrophages are both an important source of HMGB1 (33) and secrete a wide array of inflammatory mediators (34) such as MPO, IL-1β, IFN-γ, TNF-α, HnAb treatment interrupted the vicious cycle of HMGB1 release, macrophage infiltration and synthesis and release of inflammatory cytokines.

Macrophages infiltration and cytokines release also augments colonic barrier dysfunction (34), which is associated with inflammation, damage and degeneration in IBD (35). Tight junctional integrity is an important aspect of mucosal barrier function. DSS treatment caused a strong decrease in the colonic mucosal protein expression of ZO-1, claudin-5 and occluding, which was partly reversed by concomitant HnAb treatment in both WT mice and TLR4-/- mice. Transmission electron microscopic images of the DSS-treated colonic mucosa displayed loosened tight junctions and a scarcity of desmosomes, and an improvement with concomitant HnAb-treatment.

The cell surface receptors interaction with HMGB1 influence the effect of HMGB1 (36). As a damage-associated molecular pattern molecule, extracellular HMGB1 can interact with other molecules such as RAGE, TLR2, TLR4, TLR9, CXCR4, and so on (37). Our study found CXCR4 was down-regulated after HnAb application relative to IgY application in DSS-treated TLR4-/- mice. It indicated the effect of HMGB1 could not be blocked totally by TLR4 deficiency. Thus, HnAb could also ameliorate colitis in TLR4-/- mice.

Because dysbiosis is an important pathogenic factor in DSS-induced colitis, we studied the mucosa-adherent microbiome, which may be pathophysiologically more important for colonic disease than the luminal microbiota (38, 39). Previous study suggested that increased abundance of *Proteobacteria* and decreased abundance of *Bacteroidetes* were associated with DSS treatment in fecal samples (40), which was consistent with our findings. In our study, DSS treatment also resulted in a reduced proportion of *Lactobacillus* and the appearance of pathogenic *Escherichia* in the mucosa-associated bacteria. Both HnAb as well as TLR4 deficiency had a protective effect on the distribution and composition of the colonic mucosa-associated bacteria. HnAb treatment reduced the abundance of *Helicobacter*, and increased the abundance of *Lactobacillus* and *Bacteroides*. It also increased the abundance of *Ruminiclostridium*, which may helped maintain the intestinal microecology by regulating the release of inflammatory and cytotoxic factors from the gut (41, 42). TLR4 deficiency was associated with a virtually complete loss of proteobacteria and increased abundance of *Bacteroidetes* (**Figure 7**) compared to the WT counterparts. These data are in contrast to those of Xiao L et al., who found that the abundance and diversity of the colonic mucosa-associated microbiota were not affected by TLR4 deficiency (43). At the genus level, we found that the *Lactobacillus* abundance was significantly higher in the TLR4 deficiency mice than that in their WT counterparts. These data suggested that TLR4 may be involved in the regulation of intestinal microbiota. Further exploration is needed on the mechanism of regulation. Taken together, DSS-induced colitis was accompanied by severe dysbiosis in the mucosa-adherent microbiota, and the concomitant application of HnAb resulted in a shift towards the control group, both in WT and in TLR4-deficient colon. However, TLR4 deficiency was not associated with a more severe clinical manifestation of DSS-induced colitis, as has been described by Fukata M et al. (44). Possibly, the high proportion of anti-inflammatory lactobacilli in the mucosa-adherent microbiome helped to curb the colitis severity despite the absence of TLR4 signaling. The absence of TLR4 combined with the application of HnAb resulted in a particularly strong decrease of both the clinical and the histological manifestations of DSS-induced colitis.

HMGB1 also has important protective function in the colonic epithelium, because colonocyte HMGB1 was found to be directly involved in the suppression of STAT3 activation and the protection of intestine from bacterial infection and injury (45). An important protective role also exists for other cytokines that are successfully antibody-targeted in IBD flares, such as TNF-α (46). Whether the risks associated with anti-HMGB1 treatment in inflammatory bowel disease will outweigh the benefits needs to be tested in future studies.

In conclusion, the present study successfully indicated HnAb ameliorated colonic inflammation in DSS-induced colitis and TLR4 deficiency enhanced the protective effect in some aspects. It suggested that strategies against HMGB1 might provide a potential interventional approach for the treatment of colitis.

## Data Availability Statement

The raw data for 16S rRNA sequencing can be found online at: https://www.ncbi.nlm.nih.gov/bioproject/648483.

## Ethics Statement

The studies involving human participants were reviewed and approved by the Ethics Committee of Tongji Hospital, Huazhong University of Science and Technology. The patients/participants provided their written informed consent to participate in this study. The animal study was reviewed and approved by the Animal Care and Use Committee of Tongji Hospital, Huazhong University of Science and Technology.

## Author Contributions

LC, JL, FX, ZY, BS, JH, LW, YC, and MY performed experiments. LC, JL, FX, and YW analyzed data. LC, JL, FX, and US wrote the manuscript. QZ and FX provided human samples. US, DT, and FX supervised parts of the project. FX and JL designed the study and obtained funding for the project. All authors contributed to the article and approved the submitted version.

## Funding

This work was supported by grants from the National Natural Science Foundation of China (grant numbers 81470807 to FX, 81873556 to FX, 81470994 to JL) and Wu Jieping Medical Foundation (grant number 320.6750.17397 to FX).

## Conflict of Interest

The authors declare that the research was conducted in the absence of any commercial or financial relationships that could be construed as a potential conflict of interest.

## Abbreviations

Arg1, arginase 1; CXCR4, chemokine (C-X-C motif) receptor 4; DAI, disease activity index; DSS, dextran sulfate sodium; GAPDH, glyceraldehyde-3-phosphate dehydrogenase; HMGB1, high mobility group box 1; HnAb, anti-high mobility group box 1 neutralizing-antibody; IBD, inflammatory bowel disease; IFN-γ, interferon γ; IHC, immunohistochemistry; IL-1β, interleukin-1β; IL-6, interleukin-6; IL-8, interleukin-8; iNOS, inducible nitric oxide synthase; MHC-II, major histocompatibility complex II; MPO, myeloperoxidase; MyD88, myeloid differentiation factor 88; NF-κB, nuclear factor-κB; OTU, operational taxonomic unit; PCoA, principal coordinate analysis; RAGE, receptor for advanced glycation end products; RT-PCR, real-time polymerase chain reaction; T-J, tight-junction; TLR, toll-like receptor; TNBS, 2, 4, 6-trinitrobenzenesulfonic acid; TNF-α, tumor necrosis factor-α; UC, ulcerative colitis; WT, wild-type; ZO-1, zonula occludens-1.
