# Supplementary material for: Anti-High Mobility Group Box 1 Neutralizing-Antibody Ameliorates Dextran Sodium Sulfate Colitis in Mice
Source: Front Immunol. 2020 Oct 30;11:585094. doi: 10.3389/fimmu.2020.585094 (PMC7661783; doi:10.3389/fimmu.2020.585094)
Supplement: Supplementary file 1 [file Table_1.docx]

**Supplementary Table 1. Histological grading of colitis**

| Feature graded | Grade | Description |
| --- | --- | --- |
| Inflammatory cell  infiltration | 0 | Occasional inflammatory cells in the lamina propria |
|  | 1 | Increased infiltrate in the lamina propria predominantly at the base of crypts |
|  | 2 | Confluence of inflammatory infiltrate extending into the mucosa |
|  | 3 | Transmural extension of infiltrate |
| Inflammation extent | 0 | None |
|  | 1 | Mucosa |
|  | 2 | Mucosa and submucosa |
|  | 3 | Transmural |
| Crypt damage | 0 | None |
|  | 1 | Basal 1/3 damaged |
|  | 2 | Basal 2/3 damaged |
|  | 3 | Only surface epithelium intact |
|  | 4 | Entire crypt and epithelium lost |
| Percent involvement | 1 | 1-25% |
|  | 2 | 26-50% |
|  | 3 | 51-75% |
|  | 4 | 76-100% |
