# Supplementary material for: Anti-High Mobility Group Box 1 Neutralizing-Antibody Ameliorates Dextran Sodium Sulfate Colitis in Mice
Source: Front Immunol. 2020 Oct 30;11:585094. doi: 10.3389/fimmu.2020.585094 (PMC7661783; doi:10.3389/fimmu.2020.585094)
Supplement: Supplementary file 2 [file Table_2.docx]

**Supplementary Table 2. Primers used for RT-PCR**

| **Primer** | **Sequence** |
| --- | --- |
| *Human HMGB1* | *Forward: 5’-TATGGCAAAAGCGGACAAGG-3’* |
|  | *Reverse: 5’-CTTCGCAACATCACCAATGGA-3’* |
| *Human TNF-α* | *Forward: 5’-CCTCTCTCTAATCAGCCCTCTG-3’* |
|  | *Reverse: 5’-GAGGACCTGGGAGTAGATGAG-3’* |
| *Human IFN-γ* | *Forward: 5’-TCGGTAACTGACTTGAATGTCCA-3’* |
|  | *Reverse: 5’-TCGCTTCCCTGTTTTAGCTGC-3’* |
| *Human IL-1β* | *Forward: 5’-ATGATGGCTTATTACAGTGGCAA-3’* |
|  | *Reverse: 5’-GTCGGAGATTCGTAGCTGGA-3’* |
| *Human IL-6* | *Forward: 5’-ACTCACCTCTTCAGAACGAATTG-3’* |
|  | *Reverse: 5’-CCATCTTTGGAAGGTTCAGGTTG-3’* |
| *Human IL-8* | *Forward: 5’-TTTTGCCAAGGAGTGCTAAAGA-3’* |
|  | *Reverse: 5’-AACCCTCTGCACCCAGTTTTC-3’* |
| *Human GAPDH* | *Forward: 5’-GGAGCGAGATCCCTCCAAAAT-3’* |
|  | *Reverse: 5’-GGCTGTTGTCATACTTCTCATGG-3’* |
| *Mouse IL-1β* | *Forward: 5’-ACTCCTTAGTCCTCGGCCA-3’* |
|  | *Reverse: 5’-TGGTTTCTTGTGACCCTGAGC-3’* |
| *Mouse IL-6* | *Forward: 5’-GAGGATACCACTCCCAACAGACC-3’* |
|  | *Reverse: 5’-AAGTGCATCATCGTTGTTCATACA-3’* |
| *Mouse TNF-α* | *Forward: 5’-CAGGCGGTGCCTATGTCTC-3’* |
|  | *Reverse: 5’-CGATCACCCCGAAGTTCAGTAG-3’* |
| *Mouse IFN-γ* | *Forward: 5’-GCCACGGCACAGTCATTGA-3’* |
|  | *Reverse: 5’-TGCTGATGGCCTGATTGTCTT-3’* |
| *Mouse HMGB1* | *Forward: 5’-GCATCCTGGCTTATCCATTGG-3’* |
|  | *Reverse: 5’-GGCTGCTTGTCATCTGCTG-3’* |
| *Mouse ZO-1* | *Forward: 5’-GCCGCTAAGAGCACAGCAA-3’* |
|  | *Reverse: 5’-GCCCTCCTTTTAACACATCAGA-3’* |
| *Mouse Claudin-5* | *Forward: 5’-GCAAGGTGTATGAATCTGTGCT-3’* |
|  | *Reverse: 5’-GTCAAGGTAACAAAGAGTGCCA-3’* |
| *Mouse Occludin* | *Forward: 5’-TGAAAGTCCACCTCCTTACAGA-3’* |
|  | *Reverse: 5’-CCGGATAAAAAGAGTACGCTGG-3’* |
| *Mouse iNOS* | *Forward: 5’-GGAGTGACGGCAAACATGACT-3’* |
|  | *Reverse: 5’-TCGATGCACAACTGGGTGAAC-3’* |
| *Mouse Arg1* | *Forward: 5’-TGTCCCTAATGACAGCTCCTT-3’* |
|  | *Reverse: 5’-GCATCCACCCAAATGACACAT-3’* |
| *Mouse MHC-Ⅱ* | *Forward: 5’-GAGC ATCCCAGCCTGAAGA-3’* |
|  | *Reverse: 5’-CGATGCCG CT CAACATCTT-3’* |
| *Mouse RAGE* | *Forward: 5’-CTTGCTCTATGGGGAGCTGTA-3’* |
|  | *Reverse: 5’-CATCGACAATTCCAGTGGCTG-3’* |
| *Mouse TLR2* | *Forward: 5’-GTCTCTGCGACCTAGAAGTGGA-3’* |
|  | *Reverse: 5’-CGGAGGGAATAGAGGTGAAAGA-3’* |
| *Mouse TLR4* | *Forward: 5’-AAATGCACTGAGCTTTAGTGGT-3’* |
|  | *Reverse: 5’-TGGCACTCATAATGATGGCAC-3’* |
| *Mouse TLR9* | *Forward: 5’-ACTGAGCACCCCTGCTTCTA-3’* |
|  | *Reverse: 5’-AGATTAGTCAGCCGGCAGGAA-3’* |
| *Mouse CXCR4* | *Forward: 5’-GACTGGCATAGTCGGCAATG-3’* |
|  | *Reverse: 5’-AGAAGGGGAGTGTGATGACAAA-3’* |
| *Mouse MyD88* | *Forward: 5’-TCATGTTCTCCATACCCTTGGT-3’* |
|  | *Reverse: 5’-AAACTGCGAGTGGGGTCAG-3’* |
| *Mouse GAPDH* | *Forward: 5’-AGGTCGGTGTGAACGGATTTG-3’* |
|  | *Reverse: 5’-GGGGTCGTTGATGGCAACA-3’* |
| *Mouse β-actin* | *Forward: 5’-TGCTGTCCCTGTATGCCTCTG-3’* |
|  | *Reverse: 5’-TGATGTCACGCACGATTTCC-3’* |
