# Supplementary material for: Anti-High Mobility Group Box 1 Neutralizing-Antibody Ameliorates Dextran Sodium Sulfate Colitis in Mice
Source: Front Immunol. 2020 Oct 30;11:585094. doi: 10.3389/fimmu.2020.585094 (PMC7661783; doi:10.3389/fimmu.2020.585094)
Supplement: Supplementary file 3 [file DataSheet_1.pdf]

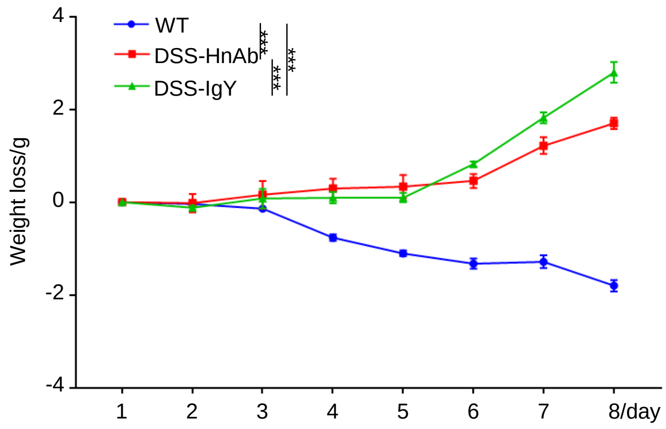

Supplementary Figure 1. Body weight loss is decreased after HnAb treatment. The body weight loss gradually increased from Day 6 onward in the HnAb-treated and IgY treated DSS-induced colitis mice. An obvious increase in body weight loss was observed both in the HnAb treated and IgY treated DSS-induced colitis mice comparing to the control at Day 8. Data were presented as mean ± SEM of 5 independent experiments. ****P*<0.001, by two-way ANOVA with Tukey's post-test.
