# Supplementary material for: Anti-High Mobility Group Box 1 Neutralizing-Antibody Ameliorates Dextran Sodium Sulfate Colitis in Mice
Source: Front Immunol. 2020 Oct 30;11:585094. doi: 10.3389/fimmu.2020.585094 (PMC7661783; doi:10.3389/fimmu.2020.585094)
Supplement: Supplementary file 4 [file DataSheet_2.pdf]

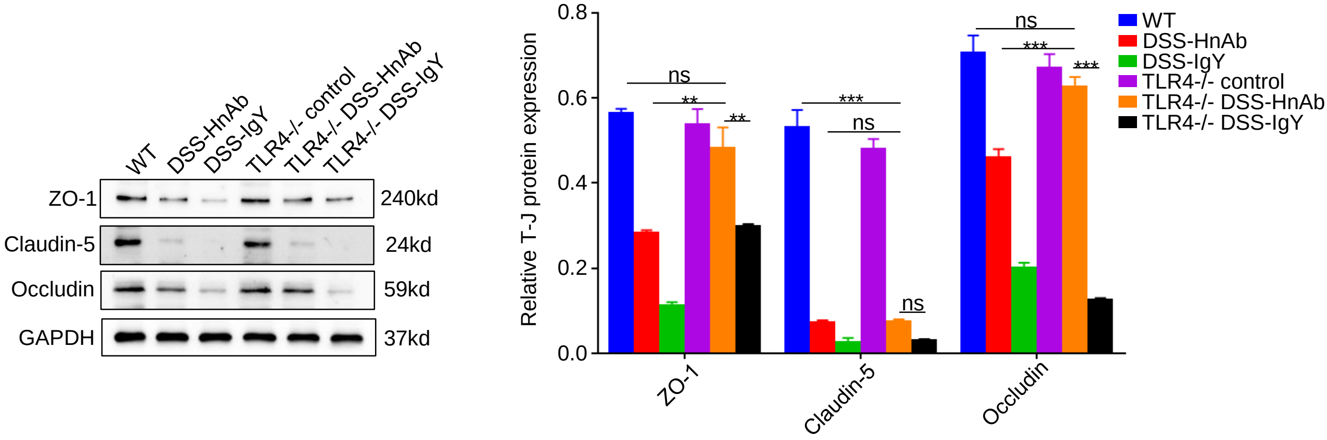

Supplementary Figure 2. Tight junction integrity of mucosal barrier is rescued due to HnAb treatment in TLR4-/- mouse model of colitis. The protein expression levels of ZO-1 (P<0.001) and occludin (P<0.001), but not claudin-5 (P=0.241), were significantly higher in colonic tissues of HnAb-treated DSS-induced TLR4-/- mice than IgY-treated DSS-induced TLR4-/- mice. A significant difference of protein expression was found in terms of claudin-5, but not ZO-1 and occluding, between DSS-treated TLR4-/- mice by HnAb application and normal control WT mice. Data were presented as mean ± SEM of 5 independent experiments. **P<0.01, ***P<0.001, ns, not significant, by one-way ANOVA with Tukey's post-test.
