# Supplementary material for: Anti-High Mobility Group Box 1 Neutralizing-Antibody Ameliorates Dextran Sodium Sulfate Colitis in Mice
Source: Front Immunol. 2020 Oct 30;11:585094. doi: 10.3389/fimmu.2020.585094 (PMC7661783; doi:10.3389/fimmu.2020.585094)
Supplement: Supplementary file 5 [file DataSheet_3.pdf]

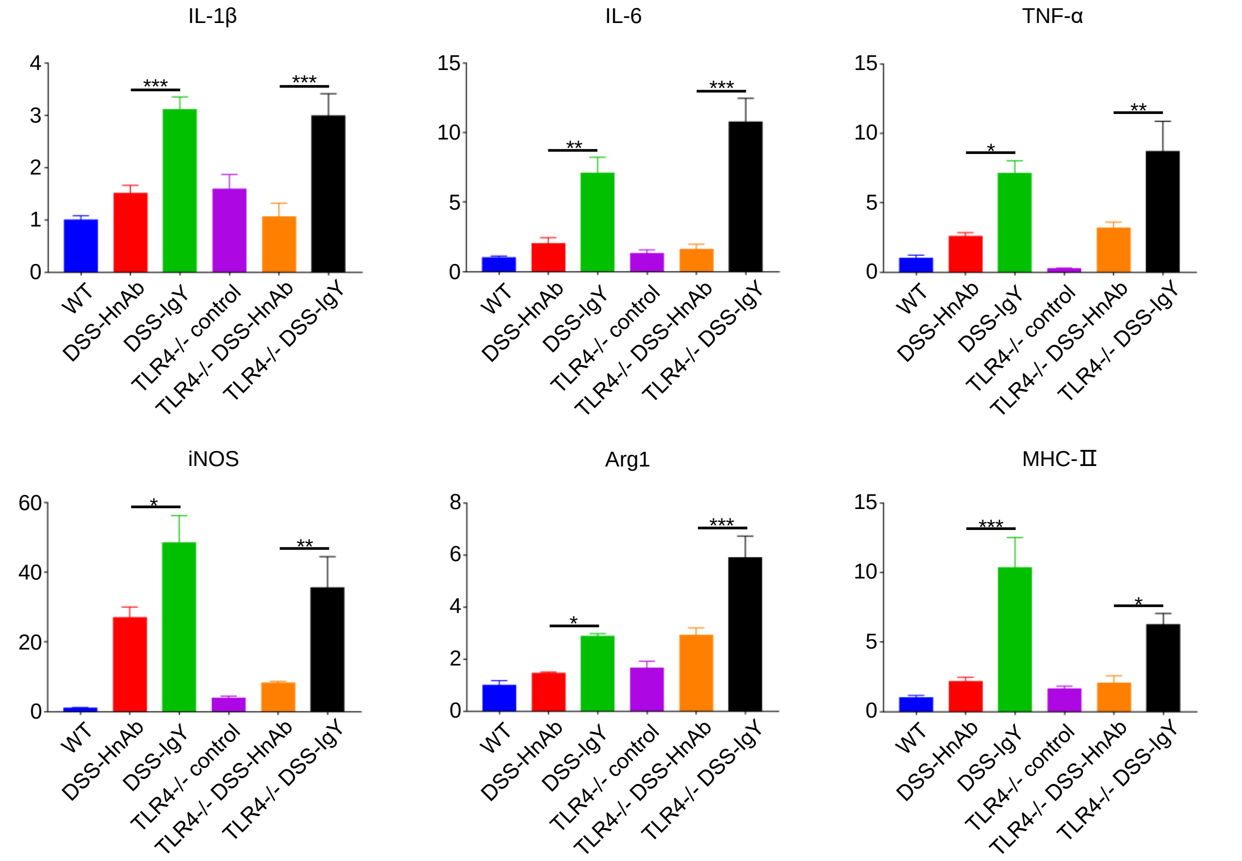

Supplementary Figure 3. TLR4 deficiency and HnAb treatment haven an effect in activation of macrophages. The expression of M1/M2 markers iNOS, Arg1, MHC-II and the pro-inflammatory cytokines TNF-a, IL-6, IL-1b was measured in colonic tissues by RT-PCR. Significant decrease mRNA expression levels for iNOS, Arg1, MHC-II, TNF-a, IL-6, IL-1b were found when HnAb treatment comparing to IgY treatment in DSS-induced colitis TLR4-/- mice or wild-type mice. Data were presented as mean ± SEM of 5 independent experiments. **P*<0.05, ***P*<0.01, ****P*<0.001, by one-way ANOVA with Tukey's post-test.
