# Supplementary material for: Anti-High Mobility Group Box 1 Neutralizing-Antibody Ameliorates Dextran Sodium Sulfate Colitis in Mice
Source: Front Immunol. 2020 Oct 30;11:585094. doi: 10.3389/fimmu.2020.585094 (PMC7661783; doi:10.3389/fimmu.2020.585094)
Supplement: Supplementary file 6 [file DataSheet_4.pdf]

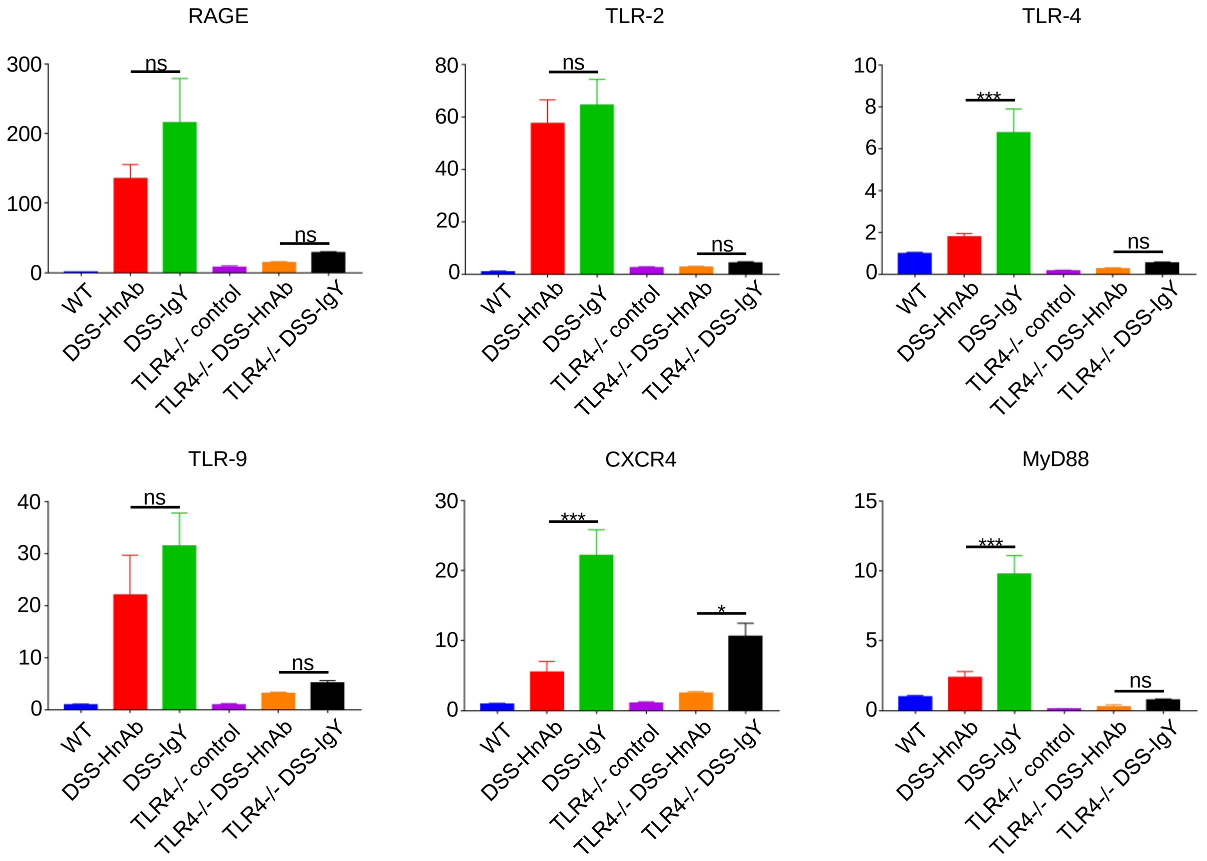

Supplementary Figure 4. TLR4 deficiency and HnAb treatment haven a different effect in the pathway of HMGB1 in DSS-induced colitis. Significant decrease for TLR4, MyD88 and CXCL4 mRNA, but not for RAGE, TLR2 and TLR9 when HnAb treatment comparing to IgY treatment in DSS-induced colitis TLR4-/- mice. Data were presented as mean ± SEM of 5 independent experiments. *P<0.05, ***P<0.001, ns, not significant, by one-way ANOVA with Tukey's post-test.
