# Supplementary material for: Anti-High Mobility Group Box 1 Neutralizing-Antibody Ameliorates Dextran Sodium Sulfate Colitis in Mice
Source: Front Immunol. 2020 Oct 30;11:585094. doi: 10.3389/fimmu.2020.585094 (PMC7661783; doi:10.3389/fimmu.2020.585094)
Supplement: Supplementary file 7 [file DataSheet_5.pdf]

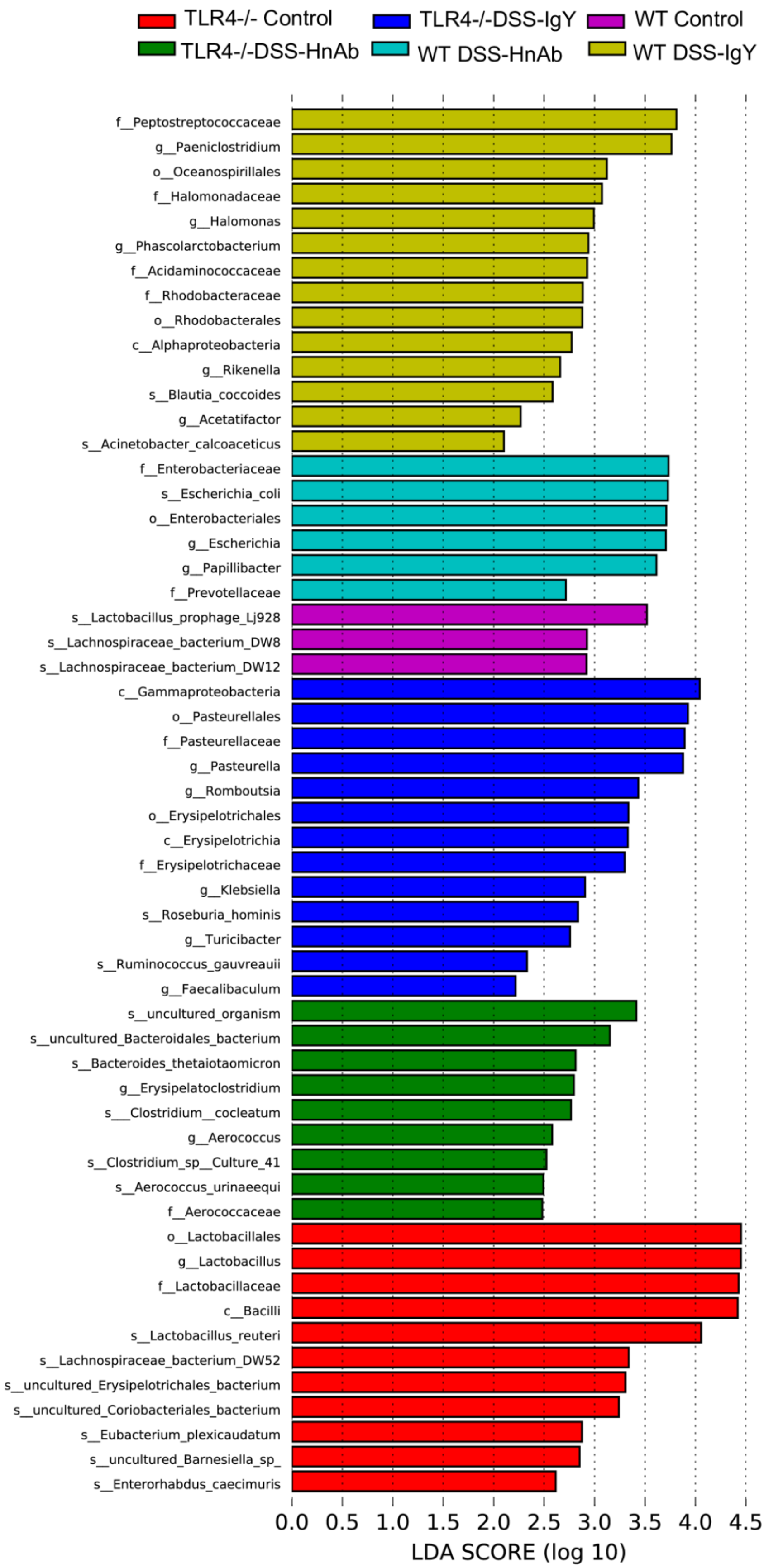

Supplementary Figure 5. Microbiota changes are partially reserved by HnAb treatment in DSS-treated mice using LEfSe. Discriminative features chart based on an effect size cutoff of 2.0 for the logarithmic LDA score were plotted. The dominating taxa in WT group were enriched in *Lactobacillus* at the genus level, which decreased after DSS administration. And the changes were partially reversed by HnAb treatment. Meanwhile, TLR4 deficiency decreased the abundance of *Bacteroides*, *Helicobacter*, *Ruminiclostridium* and increased the abundance of *Lactobacillus* compared to corresponding WT group.
